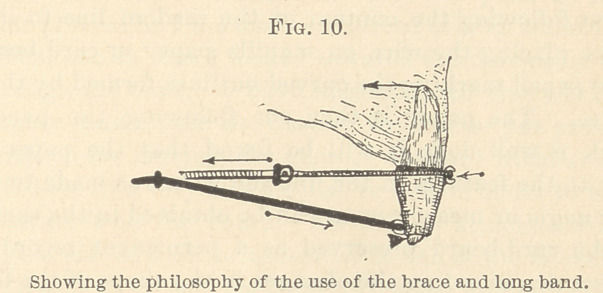# New York Odontological Society

**Published:** 1895-08

**Authors:** 

**Affiliations:** New York Odontological Society


### Reports of Society Meetings.

### NEW YORK ODONTOLOGICAL SOCIETY.

A REGULAR meeting of the New York Odontological Society was held on Tuesday evening, March 19, 1895, at the New York Academy of Medicine, No. 17 West Forty-third Street, New York City, with the President, Dr. Northrop, in the chair.

The secretary read the minutes of the previous meeting, which were approved.

### INCIDENTS OF OFFICE PRACTICE.

*Dr. S. G. Perry.*—There is a fundamental law in operation, as those who remember their natural philosophy can recall, which, stated exactly, shows that heat, light, electricity, galvanism, and magnetism increase or decrease in the inverse ratio of the distance from the central point of this source. This is a fundamental and comprehensive law which has been overlooked in the construction of our hot-air syringes.

Years ago I constructed a hot-air syringe made like a little tubular boiler, and so designed that the air had to pass back and forth about twelve times before it could escape. I used it for a number of years, but it was never very satisfactory, because, as I see now, the source of heat was too far from the point at which it was delivered into the tooth. I have thought for a long time that none of these devices really filled the bill. I think, instead of letting the air come through a large hole, it should be made to pass through a very small one, and that the heated portion of the hot-air syringe should be at the very nozzle itself. When forced through a small hole, the air comes in contact with the metal and takes up the heat, and if delivered directly into the tooth, it does not become cooled, as when it is passed through a long tube after it leaves the heated air-chamber of the syringe. I had one constructed on that principle and attached to this rubber hand-belt, as you see here. When held in the flame and warmed to a reasonable degree, and then held over the tooth, of course the current of air is steady, because it is emitted through so small a hole, and the heat is held for some time, because the end of the syringe is enlarged and made like a very large, pear-shaped finishing-bur. The next step was to protect it from the lips. Several years ago Dr. Bogue had the kindness to give me a hot-air syringe, devised by some one in Paris, with a rubber cover which could be pulled off for the purpose of heating the cylinder, and then slipped on to protect the mouth. There was great advantage in that, and I have used it for a long time. I had Mr. Drum make me a little shield of compressed paper, which is used at the present time by electricians. It is something like papier-maché, but much more dense. It is a kind of fibre. The next step was to have a nozzle made for my compressed-air apparatus. Some time ago Dr. Wassell, of Chicago, exhibited before the First District Society a beautiful hand-piece and cut-off for compressed air, and he kindly had one made for me. To this I have had one of these nozzles attached. I have never found a hot-air syringe quite so satisfactory as this device which I have here, which has a still smaller opening than the one made for the rubber hand-belt. The pressure of the cylinder just waves the flame of the lamp a little. Of course, this is all on the assumption of Dr. Brockway's idea that hot air is one of the best obtunders we have. I think it is, although it takes time and patience and care. I think I have been about as successful in obtunding sensitive dentine with the aid of hot air as with anything else.

Several years ago I called the attention of this society to a small tapering screw mandrel, constructed for the purpose of catching up a disk. It did fairly well, and I have used it with much satisfaction, and yet there have been many times when the disks did not hold as well as they should, or as well as I should like them. It occurred to me that it would be a very good plan to have a little extra thickness pasted onto the disk, in the form of a minute disk, as you see here. I had that done, making disks in this form, with a double thickness at the hub instead of the double thickness around the rim, like some disks on the market. These are picked up very quickly by the revolving tapered screw mandrel, and they hold better than with the single thickness. Of course, you can push them off if you try hard, but for ordinary purposes they do remarkably well. They will hold for all reasonable work that is done with an ordinary disk. This one is cutting on the inside now, which would be the severest test. Of course, on the other side the strain would not be so hard. But I found it was not necessary to have these disks pasted onto the large disks like the hub of a wheel. By having a block made with a depression of the exact size of the hub and of the disk, and putting the hub in first and the disk on top of it, and then plunging the revolving tapered mandrel through them both, they are instantly picked up and held firmly enough on the mandrel to do all reasonable work.

Electricity is an unmanageable agent unless handled carefully. This little syringe that I have here has a personal quality, and it is very easily managed. It takes but a short time, for the heat required is very slight. Coming through that minute opening, the heat is taken up so quickly that only a very low temperature is needed.

*Dr. Shields.*—That is one of the objections I have found. It takes such a long time to heat it. With the electrical apparatus, in a second's time you have the exact temperature you wish.

Dr. Case then read his paper on "Dental and Facial Orthopedia."

(For Dr. Case's paper, see page 463.)

DISCUSSION.

At the close of the paper, Dr. Case made the following explanatory remarks:

*Dr. Case.*—If we apply force on one point of a tooth,—say a central incisor,—in a forward direction, however near the margin of the gum we attach the power, the tendency will always be to force the crown forward, leaving the apical end of the root in its original position, or with a tendency, perhaps, to force it in an opposite direction; because the fulcrum, or that which receives the immediate force, will, under those conditions, always be the opposing margin of the alveolar socket. If, instead of that, we band the tooth and solder a rigid upright bar to its anterior surface which extends above the gum margin, and to this upper end we attach another rigid bar that is threaded at its posterior end and passed through a long anchorage tube attached to the molars, with the nut anterior to the tube, we will still force the crown forward, leaving the end of the root where it was, because the fulcrum is still the same; the power is only applied at one point a little higher on the tooth.

But now, if we attach a small wire to the lower end of the upright bar and pass it through another tube which is fastened to the same anchorage that the first one was, with a nut at the posterior end, we have something to prevent the forward movement of the crown. In other words, we have changed the fulcrum from this point in the socket to the occluding end of the tooth, and now, as we apply our force, we have complete control of our power, and can direct it to a forward movement of the entire tooth, or, as in Case VIII., oblige a much greater movement of the root than the crown.

I will now explain as minutely as I can some of the recent methods that I have used in the construction of the regulating appliances, and I will put on the board the sizes of the wire that I use. I wish to say that the entire apparatus, with the exception of the nuts, is made of German silver. I use German silver, not because it is cheaper than gold, but because I have found it to be better. There is no metal that can be made as rigid and as strong as German silver. I commence with a wire that is No. 10, Brown and Sharp's gauge; that is about .1 of an inch in diameter. That is about the only wire I buy. I draw it down to the several sizes that I use. There are three grades of German-silver wire: one is soft, another medium, and the other hard. You should use the hard, and in drawing it down do not anneal it. You can draw it down from No. 10 to No. 13; which is .07 of an inch. That is the wire I use for those strong bars for forcing forward the teeth. Where the bar extends in front of the anterior teeth it is flattened about half its diameter. The ends are threaded in the No. 0 hole of the Martin screw plate. That wire I use also for the upright bars. I also use the No. 13 wire for making the bands that surround the teeth. These bands should be very wide. The No. 13 wire rolled to .0045 or .005 of an inch in thickness will give you a band that is $\frac{3}{16}$ of an inch in width. Before rolling, anneal it, and again at different times in the rolling process. I take the measurement of the teeth in the usual way,—not from the model of the teeth, but from the teeth themselves,—drawing the ends together and soldering, being careful that no solder runs down on either side. I then usually fit it to the tooth. Then I solder on the upright piece of wire. When that is soldered, I again fit it to the tooth for the purpose of shaping the upright bar. It should fit the anterior face of the tooth. I give it a sharp turn forward at the gum margin, and then turn it back so it stands just in front of and almost lying against the gum. The upper ends of the upright bars should be cut off even with each other at the desired height, and the lower ends even with the cutting-edges of the teeth. The next thing is to cut a place for the power-bar to rest. Then I cut a groove with a small round file, at the lower end, for the fulcrum wire. Now we come to our anchorage: here also I use the wide bands on two or more of the posterior teeth. After soldering, these bands are carefully fitted to the teeth, as before, and an impression taken with bands in place. Then the bands are removed and placed in the impression, which is filled with investing material. The bands on the resultant model are soldered together where they touch, and the tubes soldered in place, without the possibility of changing their shape or position. I am particular to have the power-bar tube soldered, so that it stands in a direct line with the applied force. Also, in regard to its other relations, supposing this to be the arch on either side, I endeavor to have the tube stand so that the bar, which is a rigid one, will pass just over the cuspid tooth. If you are not particular about this it will cause you considerable embarrassment and detract from its working possibilities. In regard to the other tube below, it is not material how that stands, because, this wire being small and flexible, it makes little difference whether it stands in a line with the force or not.

If, instead of forcing the root forward, we desire to get the opposite force, I use No. 16 wire for the power-traction bar, in place of the No. 13. This is threaded in the No. 4 hole of the Martin screw-plate.

The lower, or fulcrum wire, now exerting a pushing force, should be larger than the very small wire that was before used for pulling. For this I use No. 18 wire threaded into the No. 7 hole of the Martin screw-plate.

There is one thought that I wish to impress very emphatically, and that is in regard to the anchorage. These forces are many times very great. The turning of a nut on a No. 13 wire every day is a very great force. If we did not have the fulcrum wire below, we would find that there would be considerable movement of the anchorage teeth. But, having a pushing and a pulling force attached to the same anchorage, the force at this point is largely neutralized. It is like two men standing in front of me; I push this one with my right arm and pull the other with my left. My body does not move, because the two forces are reciprocal. Oftentimes I find that the original occluding position of the posterior teeth is not changed materially, notwithstanding that the roots of the front teeth have been pushed forward considerably. You will find that to be so when you examine the models I have here, and you will find it well exemplified in Cases VII. and VIII., where the relations of the upper molars and bicuspids with the lower molars and bicuspids are almost the same in the posterior teeth, notwithstanding that the anterior teeth have been pushed forward considerably. In Case VII. no appliance was attached to the lower teeth. In Case VIII. there was an apparatus put on the lower teeth for reciprocal force, with rubber bands to the upper appliance.

In making these charts I have endeavored to give you something more than a diagrammatical drawing. I have taken the model and placed it before me, and drawn by measurement, as nearly as I could with my eye, the exact size of every portion. This I have enlarged with a pentagraph two or three times, producing the final charts before you, which are exact, as you will find when you compare them with the models themselves.

In Case I. it will be found that the cervical portion of the incisor teeth have not changed position very much. They have moved back a little. The cutting edges of the teeth have been moved forward, and the apical ends of the root have moved back considerably, carrying with them the anterior plate of the process, and thus changing the character of the facial features at this point. She had a bulging appearance of the upper lip which was quite unpleasant. The improvement to her face is inadequately shown by the models, and quite remarkable, considering the slight change, by actual measurement, that has been produced in the depression of the superior portion of the upper lip and lower portion of the nose.

*The President.*—At the suggestion of Dr. Case, the members will now have a recess of about ten or fifteen minutes to examine these models.

*Dr. Case.*—I would like to speak of Cases VII. and VIII. These are two that were exhibited at the World's Dental Congress. I have brought them with me because they particularly show the movement of the roots of the teeth, and the effect that can be produced on the face. The casts of the teeth themselves show that remarkably well. In Figure 7 I want you to lift the model off from the articulator, and look at the change that has occurred in the alveolar arch,—how the entire arch has moved forward in the movement of the four incisor teeth.

Now that the paper is open for discussion, I would like to say that if you care to please me you will confine yourselves to the points of the paper,—that is, to the movement of the roots of the teeth for the purpose of correcting facial deformities, or facial defects. I speak in this way because I have had a little experience with New York dentists. I remember quite distinctly reading a paper before a certain society of this city upon a branch of crown-work, which was confined solely to the border line between the band that surrounds the root of a tooth and the root itself. My purpose was to urge, so far as I could, a more perfect operation at that point, for the same reason that you are particular in finishing the cervical border of your fillings. Gentlemen there criticised me and expressed surprise because my paper did not go further into the entire operation, even to shaping the root; and in the discussion, instead of confining their remarks to the only point I attempted to cover, a number went into lengthy detail of various methods of crowning teeth that were entirely irrelevant to my paper.

*Dr. Guilford.*—In common with the essayist of the evening, I must say that I feel greatly honored in being invited to appear before the Odontological Society. When the invitation of the Executive Committee came to me, I did not question their right to command me or any one else to come here; but I must question their right to meet us upon our arrival, and, by feasting us, incapacitate us for speaking upon a subject as we would like to; for you all know that when the blood is drawn to the stomach it leaves the brain.

When I consented to come, it was with the distinct understanding that I should be furnished with an outline of the paper, and when that outline came it was simply a heading of four lines, which gave me little clue. Fortunately, however, I had the proceedings of the Columbian Dental Congress, and I looked over them, thinking that Dr. Case would go over the lines of his paper before that body, improving upon them, of course, and in that way I received some inkling of what he would say to-night.

I desire, first of all, to congratulate Dr. Case upon his paper and his work, because I think he has done a remarkable thing. He has taken the subject of orthodontia, which has received considerable treatment at the hands of many of our best men, and has materially improved it. He has accomplished results that I confess I never have accomplished, and which I would be very glad and proud to have done. If I take exception to anything he has said, or criticise, it is simply because I want to understand the subject a little better than I do. In the movement of teeth one of two things takes place. On the one hand we have the alveolar tissue resorbed in front of the tooth, and a new tissue formed behind it; and, on the other hand, we have a moving or bending of the alveolar plates or of the entire alveolar process. In many cases we have both. That a tooth, in moving, does not plough its way bodily through the alveolar plate is shown by the fact that after a tooth has been moved the alveolar plate is not materially lessened in thickness on the advancing side of the tooth. It did not have time to be resorbed and reformed. In the movements produced by Dr. Case, he has not only undertaken to move the alveolar process, but to move bodily forward the entire tooth or a series of teeth, roots as well as crowns, and the models look very much as if he had accomplished it. It is certainly very true that he has produced wonderful results in the improvement of facial expression. What he has done he proposes to designate by the new name of "dental orthopædia." He suggests that term in preference to the older term which we have been accustomed to use,—orthodontia. I agree with him in the statement that the word "orthodontia" does not fully express the idea that he wishes to convey by the methods which he has adopted and the results he has attained. Orthodontia is a word that falls a little short of expressing it. But the question arises, Does the word "orthopædia" properly express it? Does not that word express too much? He defined very nicely what orthopædic surgery consisted of,—the correction of deformities of the body. It is derived from two Greek words,—*orthos*, "straight," and *pais*, "a child," which means really a straightening of the child. Dunglison says that the term at best is incorrect, and that we should use words that express a straight body, because orthopædia expresses the idea not only of the correction of a deformity in a child, but also in an adult, and it is not the child that is straightened, but the bones. Consequently, we do not derive any benefit from taking a word which is incorrect and expresses too much, and substituting it in place of another word which falls a little short in its meaning. While it does not exactly express the idea we wish to convey, the word orthodontia comes nearer to doing so than any other, and hence seems to be the better word to employ, for when we correct facial expression we do it by the movement of teeth. In regard to the movements that have been spoken of by Dr. Case, we must remember that the alveolar process is built up on true bone, and it is thickest where it is attached to the bone. As that extends downward it gets thinner and thinner, until it terminates entirely at the neck of the tooth. At this point there is less bony tissue to be moved, and it can be more readily moved than where it is thicker. The apex of the root is nearly stationary in ordinary movements, but as we move the crown we must overcome the resistance of the alveolar process at its margin where it is thin, midway where it is thicker, and at the apex where it is thickest. When we undertake to move a tooth bodily, forward or backward, we have the resistance all along the line of the root, and we have the greatest resistance at the apex of the root, because there the alveolar process is thickest. To move the apex of the root any considerable distance would require the expenditure of a great amount of power, and probably necessitate the moving of the maxillary bone as well. Can this be done? That Dr. Case secures a movement at the end of the root I believe, but that he accomplishes it to the extent that he thinks he does I very much question.

In all the cases represented here, with possibly one or two exceptions, you will notice that where he has moved the teeth of one jaw, he has also moved the teeth of the other.

In determining the amount of movement that we get, or the real effect of our operation in the movement of the teeth of one jaw, we ought to have a fixed point to calculate from. When you move the teeth of both jaws, one in and the other out, you remove the basis of comparison, and do not know exactly what you are getting. We do not know how much the one has moved in and the other out. We get the improvement in expression, but it is a question whether we have moved the roots of those teeth as much as would appear. While, therefore, Dr. Case has accomplished wonderful results, there is a question in my mind whether he has not overestimated the amount of motion that he gets along the entire length of the roots of the teeth in many cases. In every extensive case of irregularity that we correct, where we propose to move the upper teeth or the lower ones, we get an improvement in facial appearance that is very considerable. If the results are not as marked as those shown to-night they are nevertheless remarkable. I have often been surprised at the change in facial expression brought about by the movement of the teeth. I suppose we all move the roots of the teeth more or less, but I doubt whether we change them as much as Dr. Case would have us think.

I brought the models of some jaws with me to-night which prove the point I spoke of. In one case where there was marked protrusion of the lower teeth I decided to do two things, the first of which was to retract the lower jaw. I used a skeleton cap for the head and a metal and leather chin-piece, with rubber straps connecting the two. In addition, I employed a vulcanite plate with spurs of gold fitted to the roof of the mouth to assist in guiding the lower incisors into place. This was kept in place by platinum bands attached to the molar teeth with lugs at each side. In this way it was held in position while the lower teeth bit upon it. After using this chin-appliance for a while, I seemed to have accomplished a very wonderful result; but the gain ceased very soon, and I had to try something else. I extracted a bicuspid on each side, and retracted the six inferior anterior teeth. In the end I secured a very perfect occlusion, as you see.

While the deformity in this child's face was very marked indeed, as soon as the upper teeth were brought out and the lower ones in, the entire expression was changed and harmony of the features restored. I did not try to move the apices of the roots of those teeth, and yet I secured as perfect results as I could have wished for. Again, I desire to give Dr. Case full credit for what he has done, which is certainly a great advance upon previous methods, and at the same time to thank him for his splendid exhibition of masks, casts, and appliances.

*Dr. Bogue.*—Before undertaking to discuss any portion of Dr. Case's paper, I should like to express here a hope that Dr. Guilford will show us all, one of these days, the models (both before and after) of a retracted set of fully erupted lower teeth where the bicuspids have been extracted, and the teeth anterior to that space so formed have been retracted. With a pair of compasses in my hand, I should like to examine such a case.

*Dr. Guilford.*—There is one right here, Dr. Bogue, which you may examine at your leisure. This is a case where the two bicuspids were extracted before either they or their neighbors had attained their full size or assumed their rightful positions in the jaw.

*Dr. Bogue.*—It has been claimed that the apical ends of the roots of teeth do not move from their original locations, but when Archimedes discovered the identity of the inclined plane with the wedge, wheel and axle, and screw, he exclaimed that if he had a place to stand he could move the world. Dr. Case has apparently found a place to stand. I expect to see him move the dental world, as well as the apical ends of the roots of teeth. But, as we are only to discuss this evening the operation of his instrument on the six front teeth, we are far from settling the question of orthodontia in general. While I have unstinted praise and admiration to offer for Dr. Case's admirable invention, and believe it the most efficient and at the same time the simplest instrument thus far devised for its purpose, I will beg the gentlemen present to notice carefully that Dr. Case does not enter into the discussion of, nor even touch upon, the subject of the proper occlusion of the grinding surfaces of the molar and bicuspid teeth, upon which depends, in a large degree, the ability to masticate, and hence to prepare for the digestive process all food taken into the mouth. Leaving out of view, therefore, this matter of occlusion, where, possibly, I might have some sharp differences of view with the essayist of the evening, I will confine myself entirely to the discussion of his means of beautifying a countenance more or less deformed through the malposition of the six front teeth. In all orthopædic surgery the question of bending the immature bone of the growing child has to be considered, and its advisability decided upon, and then means are adapted to the end desired. Now, whether it be ring-bone in a horse, or certain morbid growths on the bones of the human body, or the change in location of a child's tooth, a positive pressure which is periodically repeated, with intervals of rest between, is, I believe, the best mode yet discovered for accomplishing that object. Dr. Case has recognized this principle, and has adopted the screw as his motive power. The screw, unlike the elastic pressure of a spring, can be made to give a definite advance by means of definite pressure, and then stop, that nature may recuperate until the time arrives for another application of pressure. Another point needing to be carefully noticed in this discussion, and in all action growing out of a necessity for these operations, is the age at which the roots of the teeth attain their full size. This question has a deep bearing on all that Dr. Case does for children, and it will go far, I think, to explain some occurrences that take place in his operations for adults. If the skull of a child of six years be examined by dissecting away the anterior plate of alveolus covering the permanent teeth that are just about to erupt, it will be found that the roots of the first permanent molars whose crowns are entirely through the gum are not fully grown, and consequently have no apices; so that if the bodies of these teeth are moved to any position different from the location in which the crowns erupted, the further growth and completion of that root will take place while the tooth is in that position. This is still more true of the other molar, bicuspid, and cuspid teeth, the crowns of all of which, excepting the wisdom-teeth, are in the skull at that age.

The main point which occurred to me in discussing the question with Dr. Case was that perhaps none of us had sufficiently examined into these questions of development which, under these circumstances, seemed to me to be of paramount importance. It will be seen with a moment's thought that the exact age at which each tooth is completed is a matter of very great consequence when we apply fixtures that move the crowns from one position to another, and still more must we be positive of the time of completion when we undertake to move not only the crowns but the roots bodily, and to endeavor to have that action reflected in the alveolus and the outer integuments of the face. I did not want to do much more than express my thanks for the apparatus which has been shown, and in saying what I have I must beg the pardon of the gentlemen for not having been able to go into the matter more carefully.

*Dr. Farrar.*—This society should congratulate itself upon having such an interesting presentation of this subject as the essayist has given us this evening. Now, to his question, do we "believe that the bones around about the roots of the teeth were bent?" I will say I think they were; but just how far they were bent, or just what and where all the parts were altered, of course can only be inferred from the outward appearances. If we could take the flesh off the bones before the operation, and then take a cast of the bone, then return the flesh, regulate the teeth, and then take another cast, we could prove the facts; but at present we must be content with reasoning upon the subject. That the bones under the lower part of the nose can be bent by applying force to the incisors I have had proof of in some of my operations. Let me tell how I first caught upon the fact. Several years ago (1886 and 1887) I had occasion to regulate a case of protruding upper teeth for a young lady about fourteen years of age; the lower part of her lower lip was somewhat prominent, but the upper part was not as full as it should have been. I drew the lateral incisors posteriorly (after having caused the cuspids to naturally move into the places of the extracted first bicuspids) by a gold skeleton mechanism anchored to the posterior teeth, and when these teeth were in their proper places (against the cuspids) the same mechanism was applied to the centrals; but the anchorage resistance was not sufficient to move them far without moving the posterior teeth forward, therefore I was obliged to resort to the skull-cap or head-gear, a sort of harness, having gold draught wires connecting it with the ends of the crowns of the incisors. By retightening this harness these teeth (crowns) moved posteriorly, in the same way as other dentists have noticed in their operations; but now comes the point intended by mentioning this case. One day the father called on me and said, "Myself and wife have concluded to take our family to Europe; now, how soon can you push my daughter's case through?" He also told me when he would like to sail. I replied, "It can only be hurried by increasing the force, and perhaps the case will not permit of great increase without causing too much pain; but I will immediately begin the trial." The draught upon the teeth was increased gradually, and in a few days it was carried to a point that caused the head to ache slightly. Shifting lower a part of the anchorage so as to include the base of the occipital region, the full force was maintained. During all this time, however, the patient said that she noticed no pain about the teeth, but incidentally remarked that there was a peculiar feeling just under the nose. This, however, made no impression on my mind, as I had often heard similar remarks from other patients under similar operations.

We were now applying all the force that the harness would permit without causing headache, and the teeth were moving gradually,—but not as rapidly as the parents desired. To my surprise, however, the father and daughter called one evening about ten o'clock, and said, "My daughter's teeth are now moved in far enough." I examined the case, and sure enough the teeth had moved more rapidly than I ever knew teeth to move before, and had reached their proper places. But what had caused the sudden change seemed mysterious until I examined the contour of the entire lip and nose, and found that the same changes had taken place that Dr. Case's casts present. The upper part of the lip was now filled out, and the end of the nose was slightly advanced.

*Dr. Case.*—Were the nerves destroyed?

*Dr. Farrar.*—No; nor were they injured. It was plain to be seen that the drawing upon the ends of the crowns had thrown the roots forward, and that this was the cause of the outward changes in the lip and nose; but whether the suture between the halves of the upper jaw-bone had yielded and the borders of the bone turned outward, or whether sufficient decalcification had taken place in the bone to enable it to bend by the leverage of the teeth upon it, I could not determine; but one thing was certain, great changes had taken place in its contour, and that the roots of the teeth had moved forward *en masse* by tilting on fulcrums at the necks. (See *F* in Fig. 1.) This was a lesson that led me to an idea of the possibilities of such operations, and I immediately determined to work upon this line with improved mechanisms. I now have some half dozen, all based upon philosophical laws. I have brought with me several engravings of these, taken from my forthcoming volume, which I will pass around after I have sketched them upon the blackboard, so as to explain their action. I wish to say, however, before I proceed, that I regard Dr. Case's mechanism not only simple but philosophical; that it is practicable he has proven by his results. Mine differs from his, and, therefore, the combination of his mechanism belongs to him.

Fig. 1.

Showing the changes made in the position of the teeth.

As you will finally see, the engine of force in all of my mechanisms for moving roots *forward* are placed within the dental arch, instead of outside of it, as in the essayist's cases. In the mechanism represented by this sketch (Fig. 2) the base of support is a trans-

Fig. 2.

Mechanism for moving the roots forward *en masse*. (Farrar.)

palatine screw-jack, anchored by two clamp-bands, that embraces the side teeth ; from this jack to the posterior sides of the necks of the incisors, and lying close to the sides of the arch, are two other screw-jacks to press against these front teeth; to hold these jacks upon them, each incisor has upon it a broad ferrule (cemented), with a U-shape lug on the lingual side, near the gum (see *F* in lower part of Fig. 2), in which a bar connecting the anterior ends of the jacks rests. To hold firmly the ends of the crowns of the incisors, and prevent them from moving forward when these jacks are set at work against the necks of the teeth, they (the ends) are tied to the transpalatine jack by two wire cords, connecting with a cross-bar lodged in other U-shape lugs soldered to the labial side of the ferrules, near the ends of the teeth, as represented by this sketch. (See Fig. 3).

Fig. 3.

Showing the principle of applying compensating force.

In another mechanism I use more radial screw-jacks than in this one, for forcing the roots forward; these are arranged thus (see Fig. 4). The ends of the crowns are held fixed by a wire bow (see Fig. 5) placed in U-lugs (see dotted line in Fig. 4), one being hooked into a wire ring soldered to the lingual side of one of the anchor-bands, and the other screwed to the corresponding side of the other band. (See *A A*.) It is a modification of my screw long band.

Fig. 4.

Radial screw-jack mechanism, for moving the roots of six teeth forward. (Farrar.)

Fig. 5.

The check-bow belonging to the radial mechanism. (Farrar.)

Another and similar mechanism is made like this sketch (Fig. 6). It consists of two anchor-bands (*B B*), a transpalatine screw-

Fig. 6.

Mechanism for moving forward, *en masse*, the roots of the upper incisors. (Farrar.)

jack with two hooks (*H H*), four ferrules (*F F F F*), a pushing bow (*P*), operated by nuts (*N N*), and stay- or check-cords (*D D*), with a cross-piece.

*Dr. Jarvie.*—What are they for?

*Dr. Farrar.*—To hold the crowns and to throw the roots of the incisors forward.

This mechanism is operated by four nuts (N N, N′ N′), two to push the bow (P) forward, and two to draw the crowns backward. The extremities of the arms of these rods play in smooth, bare tubes and nuts, as shown here.

This next figure represents another mechanism for the same purpose. (See Fig. 7.) It consists of two anchor-bands, two screw-

FIG. 7.

Mechanism for moving forward the roots of upper incisors *en masse*. (Farrar.)

jacks soldered to a cross-piece, four ferrules (F), and a wire bow. The main difference between this mechanism and the first one described (Fig. 2) is that there is no transpalatine jack, the anchorage of the two jacks being made directly upon the anchor-bands, as shown.

As will be seen, all these mechanisms are for moving *forward* the roots of the front teeth where the upper part of the upper lip

FIG. 8.

Representing an example of the class of cases that require the moving forward of the roots of incisors.

is sunken, as represented by this sketch (Fig. 8); but for moving the roots posteriorly, as needed in cases where the upper part of the lip is too prominent, they would not be practicable without some modifications being made in them; these modifications can be made easily. This mechanism, like all the others that I have described, acts compensatingly, one force upon the anchorage being balanced by the others.

The following sketch (Fig. 9) represents a mechanism designed for this purpose, and it is very similar to several that I

FIG. 9.

Mechanism for moving posteriorly the roots of upper incisors. (Farrar.)

published many years ago. The crowns are stayed by an inside rectangular frame, resting in U-shape lugs at the ends of the crowns, and braced against nuts soldered to two anchor-clamp bands on the side teeth; the roots are drawn back by what I call

FIG. 10.

Showing the philosophy of the use of the brace and long band.

a screw-acting long band resting across the labial sides of the necks of the teeth to be acted upon and attached to the clamp bands by screws. (See Fig. 10.)

Now, in regard to Dr. Case's beautiful presentation of face-casts. I wish to compliment the essayist upon his ability to secure the privilege from his patients of obtaining them. I am seldom successful in obtaining even a photograph, much less a cast. Whether the people in New York are prouder than they who live in the West, I do not know, but could I have the privilege given to Dr. Case I think I could present some very interesting cases in the line of orthodontia, but nothing more interesting in this line than Dr. Case has given us.

*Dr. V. H. Jackson.*—The subject that Dr. Case has brought before us I have been interested in for several years, and would like to call attention to a paper I read before the Odontological Society in 1887, in the discussion of which I stated, " At some future time I will describe my method of carrying the incisors forward bodily without changing their angle" (*Dental Cosmos*, vol. xxix., page 385), and since then I have been interested in moving the teeth bodily; but then it was not accepted by the profession.

Dr. Case has proven in this and previous papers that it is possible, and from my experience I can say that I have practised for several years moving the roots of teeth in the direction desired.

I want to congratulate Dr. Case in presenting the matter in such a delightful manner as he has done with the models of the face. I have adopted a system of making measurements of the face and also the contour, which I think will be of service to the gentlemen present. I will explain that before describing my method of moving teeth bodily. My method of making a permanent record is to take a piece of soft lead or tin wire about one-eighth or three-sixteenths of an inch in diameter (the square is usually preferred), which is shaped to the contour of the features, usually first following the contour of the median line to show the profile, then placing the wire on manilla paper or card-board, and with a fine pencil marking the curved outlines formed by the shape of the wire. The paper is then cut following the pencil line, if the work is well done it will be found that the paper will fit accurately to the features in the line the wire was made to assume. Any other angle or measurement can be obtained in the same manner, and the card-board preserved as a permanent record, which will be of service in noting the changes in the shape of the features, either for immediate use or to note future changes.

Dr. Jackson demonstrated with a patient the method as described.

It is tedious work to make plaster casts of the face, and I would like Dr. Case to explain to us his method of making them. I have recently used modelling compound more or less for this purpose by rolling it thin with a rolling pin, and placing it upon a piece of thin sleazy cloth that is capable of changing its form. It is then softened by holding either end of the cloth and running it through a warm-water bath. This is then applied, the patient having become accustomed to the heat by the application of cloths wrung out from warm water. I have found that pressure in applying the modelling compound will occasionally push the soft tissues out of shape, especially the nose and lips, and will not give a correct reproduction of the features; consequently, I frequently cut a hole in the centre of the compound and cloth sufficiently large to pass over the nose and mouth, and, after adjusting it, introduce into the nose a quill wound with cotton, so that it shall not interfere with the breathing, and cover the portion of the face that is exposed with plaster of Paris. In most of the models that Dr. Case has presented the patient had the advantage of breathing through one nostril, as only one side of the face was taken. There are several deviations from the method described which I have practised. I think the plaster of Paris gives a better effect than the compound. A thick roll of compound may be preferable for making a model in certain cases.

With regard to moving teeth other than superior incisors bodily, in the discussion of a paper read before the First District Dental Society at an anniversary meeting, in speaking of the results of spreading the arch, I described that the teeth (molars and bicuspids) are often elongated by the resistance of the true bone of the malar process of the superior maxilla, which "often complicates the operation of spreading the arch, as it does not yield as readily as the alveolar process, and the teeth are forced over it, which elongates them." That shows that I looked towards the moving of the roots bodily through the bone. I have accomplished this work in a different manner than either Dr. Case or Dr. Farrar has presented.

One method for moving the incisors outward without changing their angle is to fit a very broad collar to each of those to be moved. I will illustrate it on the board. An arm is attached with solder to the lingual side of a collar cemented to one of the lateral incisors which is formed to extend back, following the lingual curve of the teeth to the second or third molar. The effect of this would be like fastening a collar with an arm extending from it around the end of a cane and pulling on the cane; pressure would be caused on the whole length of it, providing you have a stiff arm to support it. Here would be the root and here the crown. A short arm is then soldered to the lingual side of the collar on the central incisor, and made to form a gentle curve to and is soldered to the arm that extends to the distal part of the arch. A similar long and short arm is soldered to the collars on the other lateral and central incisors which extends back on the other side. A crib is then made over a bicuspid and molar on each side of the arch for anchorage. A base wire is arranged to cross the arch following the palatine curve, passing over the arms described, and is soldered to the crib portion on each side. Tubes can be attached with solder to the crib portion underneath the base wire through which the arms can pass, preferably with the under side of the tube cut away. A long U-shaped loop of spring wire should be formed for each side of the arch, one end of which should be soldered in front of the base wire near the tube; the other end should project a little above, and made to catch into a hook that is soldered to the arm that extends back from the incisors. The necessary pressure to move the incisors outward is caused by opening the loops by bending and springing them into the hooks that are attached to the arms. A similar appliance is constructed for moving the ends of the roots inward, by reversing the hooks on the arms, and having the looped spring arranged so as to pull inward rather than to push outward as described.

With regard to the movement of the roots of incisors, I have had results that have led me to believe that the premaxillary bone has been moved forward or has assisted in compensating with the pressure that is against it. Some of the sutures do not unite.

I would like it to go on record that I have called attention to the consideration of the shape and size of the nose before regulating the teeth. I have spoken of it for several years in class work, etc. The shape and size of the nose should always be studied in connection with the features before regulating, and especially to determine whether to move the superior inward or to move the lower teeth outward.

I am glad that Dr. Case has brought before us such beautiful specimens and charts.

[Note.—Dr. Jackson intended to describe the following appliance (but was called to order for lack of time) which he had devised for moving the incisors forward bodily, all of which, except the collars, can be removed for cleansing. It was made by soldering a tube or socket in the palatine surface of the collars that were cemented to the incisors. A heavy base-wire was then formed in the usual manner, with short arms soldered to it in position to project into the tubes soldered to the collars. The other portion of the appliance was made similar to the one described.]

*Dr. Case.*—I have been exceedingly gratified at the kind remarks which have been made on my work by men of great eminence who are here to-night, and it has given me much pleasure. I look upon this work somewhat as I would on a child that I am bringing up and introducing to the world. I feel grateful to you that you have shown me the compliment of asking me to come here and represent things just as they have occurred in my practice. That is a great deal. In Detroit, the other day, I listened to a poem the sentiment of which was something like this: In our youth the greatest thing we strive for—the golden apple that we seek to pluck from the tree of time —is fame, followed, possibly, by wealth ; but as the years go on we come to understand that it is greater to be loved than to be honored ; greater to be trusted than admired. And that is the way I feel to-night,—that it is greater to be trusted than to be admired, because there are so many opportunities where one could deceive. I want this child to come up before the world without any deception whatever, and I have brought these models here because I believe they exactly represent the changes that have taken place. How a man like Dr. Guilford can examine some of these models,—for instance, the model of Case VII.,—and look at the change that has taken place forward of the cuspid teeth, leaving the articulation of the posterior teeth exactly as they were, and say it is any different from what it seems to be, strikes me as very strange indeed. There was nothing put on that lower jaw at all. In Case VIII. there was no change to the lower jaw. You can examine the jaws of all these teeth, and you will find no special change to the lower teeth. As far as the articulation is concerned, that can be adjusted in time, if it is broken up.

In regard to the change in features of the face, that is dependent upon more than a movement of the teeth and alveolar process. The bones of the face are similar to other bones, and are undeveloped and largely cartilaginous until a certain age. When we apply force to the teeth in this way, the teeth are merely used as places for attaching the force appliances; so that the force is directed to a movement of the entire bone in which those teeth are embedded,—not the process alone, for, it strikes me, it must go farther than that, in order to affect so much of the middle features of the face. Case I. shows the effect upon the nose, which was decidedly *retroussé*,—and turned up at the end. It was brought down and straightened. I have a number of cases in my office where this is particularly exemplified. In Case II., you see, the nose is changed from being turned down at the end to a straight nose. In Case III. the nose was made straight at the end. Case VIII. was a young lady, a Jewess, with a nose characteristic of that race, but I made a Yankee of her, as I told her. I had believed, and I do believe, that force applied in this way affects not only the alveolar process, but the shaping of the bones of the face.

In regard to casts, or models of the face, it is an easy matter for any one who has the proper individuality, to get an impression. If you are going to construct an artificial denture, you simply say you must have an impression, and you get it. You do not say, "Please allow me to take an impression of your mouth." You say, "We are now ready for the impression;" and if the patient says, "What! are you going to take an impression of my face?" you say, "Yes, of course; it is my guide."

It is a simple thing to take an impression of the face in plaster. I wish I had the opportunity to show you how easy it is. It takes less than ten minutes from the time you commence putting on the plaster to the time it is off the face. I have often had my son time me from the time we commenced to put on the plaster until the removal of the cast, and it often has taken no longer than six minutes. It does not inconvenience the patient very much. I will describe the method as nearly as I can. I have two bowls, to commence with. I say to my son, who assists me, "I am going to take a face-impression." He fixes the water in both bowls; puts in sulphate of potash, the right amount to make it set rapidly; then I have a bowl of clean white vaseline, with a clean brush. That congeals in the ordinary temperature. It is put into a bowl of hot water until it becomes liquid. The bowl is brought to my side. In the mean time I have composed my patient, told her just what I am going to do, first covering the face with vaseline, which I proceed to put on the face, talking to her all the time. Where it goes over the eyebrows, or where there is considerable hair on the face, I go over it twice or more. One of my patients told me the other day she could never keep her face straight, and I told her to laugh all she wanted to. Had I told her not to laugh, she would certainly have done it. I turn the patient on one side or the other, just as is most convenient. I compose the face, having gone over it with the vaseline, telling them to see that the teeth are closed in the masticating closure, and telling them to keep the mouth and face still; then I say to my young lady assistant that I am ready for my first bowl of plaster. My son fixes that, she brings it to me, and I lay it on with the spatula, beginning with the mouth, because I want that most perfect. If the patient does laugh, I can take it off and start again. I carry it back as far as I can, and when it commences to harden I call for another bowl. I go right on, having the patients open their eyes while the plaster is being carried close to the lower lid, then over the ear, having a little cotton in the ear,—and that has probably finished two bowls. Over the face itself the plaster is about one-fourth to one-half of an inch thick. The next bowl of plaster is carried over the forehead, eyebrows, and upper lid of the eye, which at this time is closed. Then there is a little slit left there that is slightly open; you can see by the models I have here. I have used possibly three bowls by this time, and then another one is brought. The last one I order with enough sulphate of potash to set very rapidly. Then I bring that over the nose, and meet the other parts of plaster. You do not put anything in the nose. You simply manipulate your plaster right off the end of the spatula, so the nostrils are left open, and you do the same when you take the entire face. As soon as the plaster commences to harden you will have a syringe of cold water to throw on. You must keep it wet and cool while it is hardening. When sufficiently hard, tell your patient to move the muscles of the face a little; get a slight tension on the borders of the impression,—not too much,—and then it all comes off in one piece. It never takes over ten minutes; generally I get it in six minutes. It is a very simple thing, and after the patient has gone through the process once you can go through it as often as you like for that patient.

*Dr. Guilford.*—I am very much afraid that Dr. Case misunderstood me, or else that I did not express myself clearly. The principal idea I meant to convey was that in the cases shown here, with the exception of the two he mentioned where the lower jaw was not operated upon, having moved both the upper and lower jaws, he was perhaps mistaken in the amount of movement he had secured in a single jaw, and that he was giving one jaw credit for greater movement than had actually occurred.

The thanks of the Society were tendered to the essayist and the gentlemen who took part in the discussion.

Adjourned.

John I. Hart, D.D.S.,
*Editor New York Odontological Society.*

## Figures and Tables

**Fig. 1. f1:**
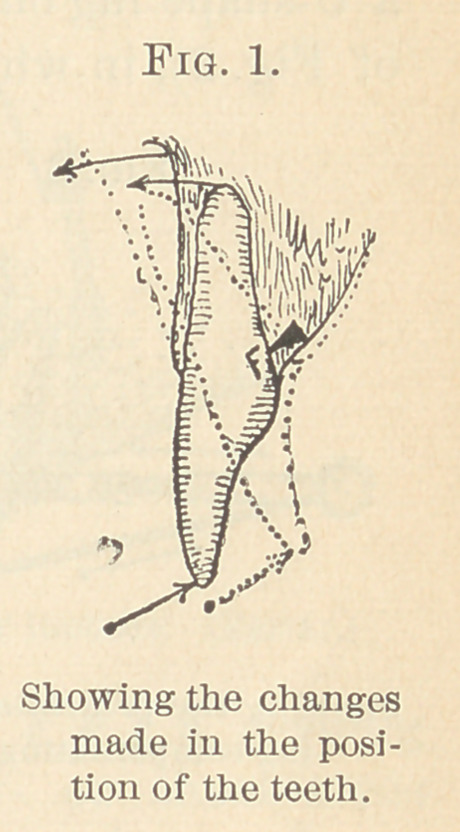


**Fig. 2. f2:**
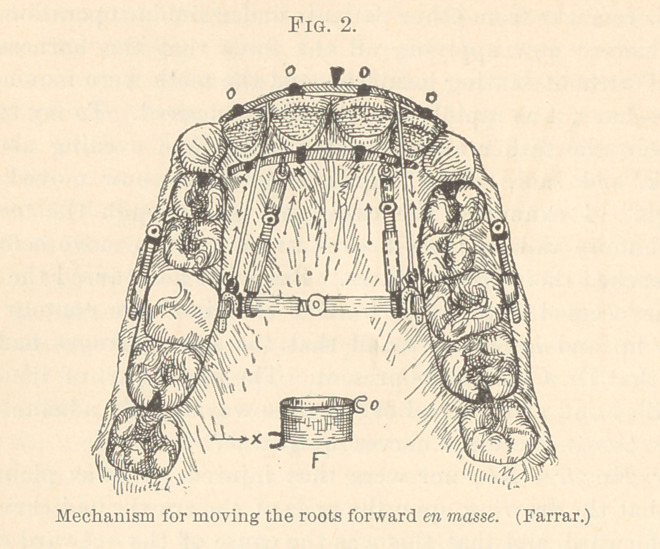


**Fig. 3. f3:**
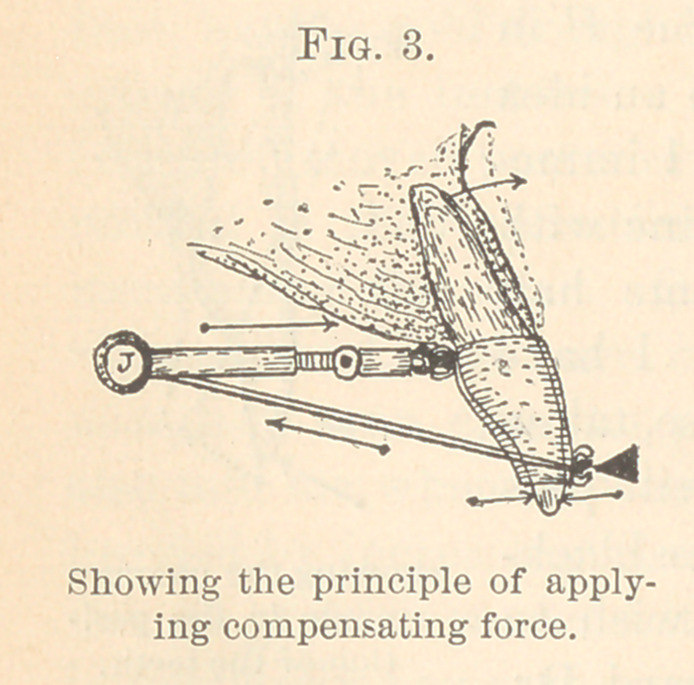


**Fig. 4. f4:**
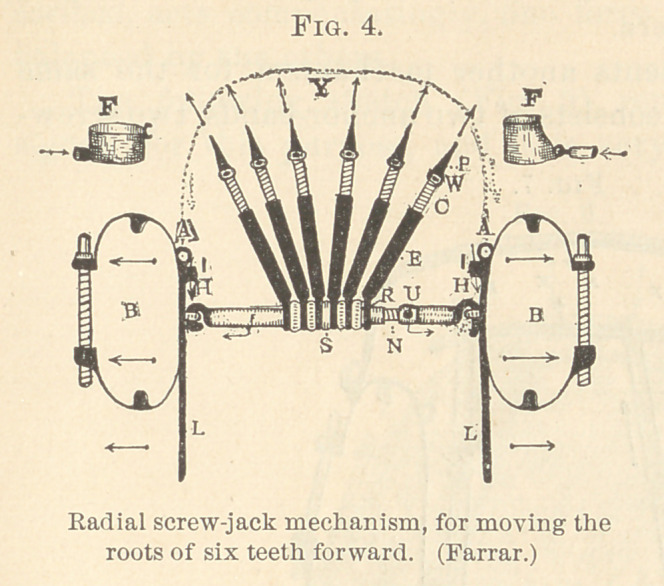


**Fig. 5. f5:**
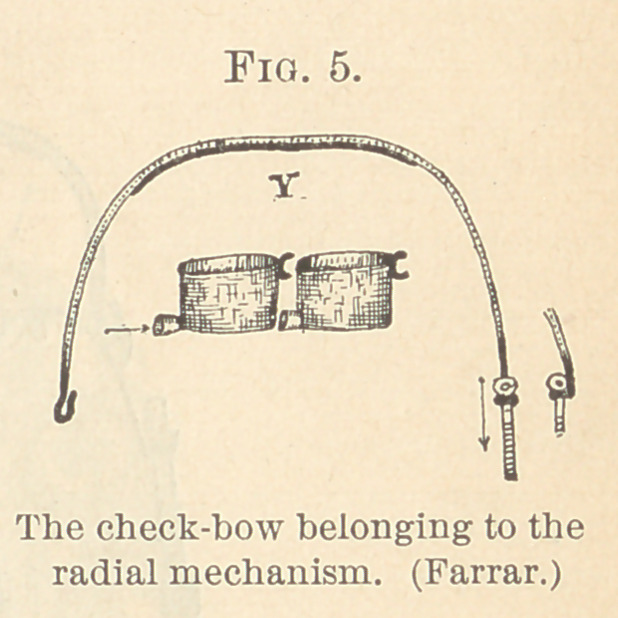


**Fig. 6. f6:**
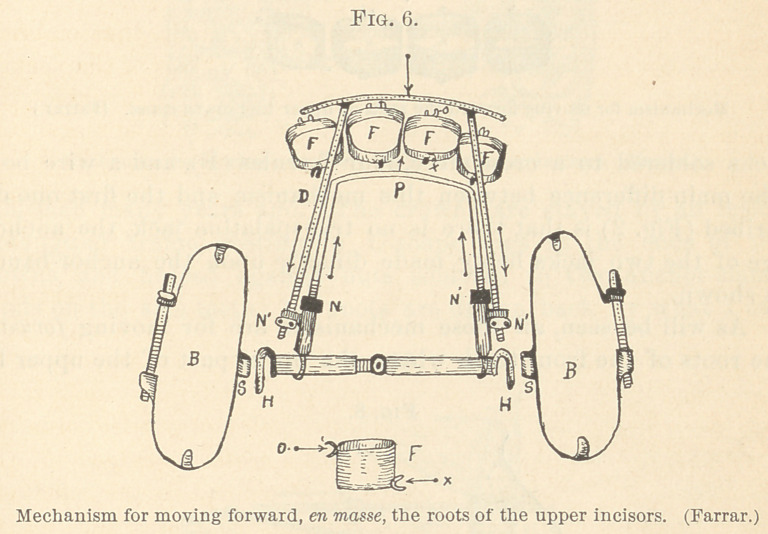


**Fig. 7. f7:**
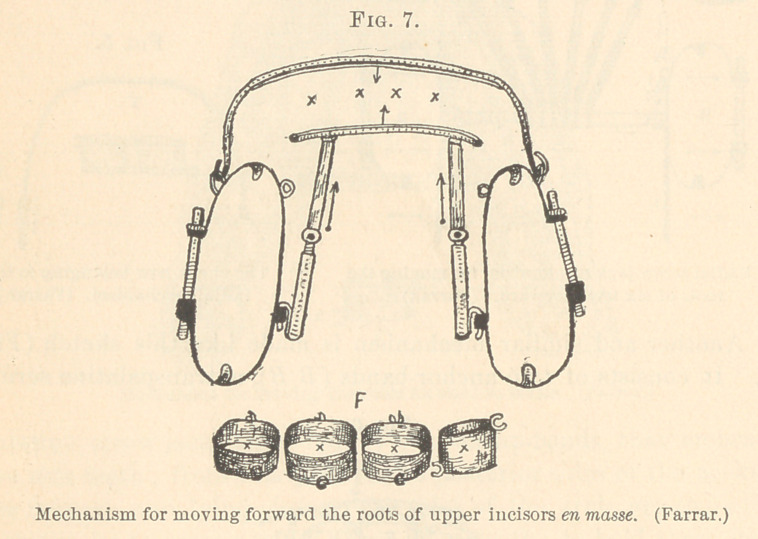


**Fig. 8. f8:**
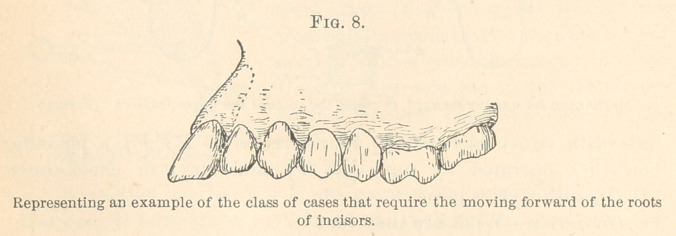


**Fig. 9. f9:**
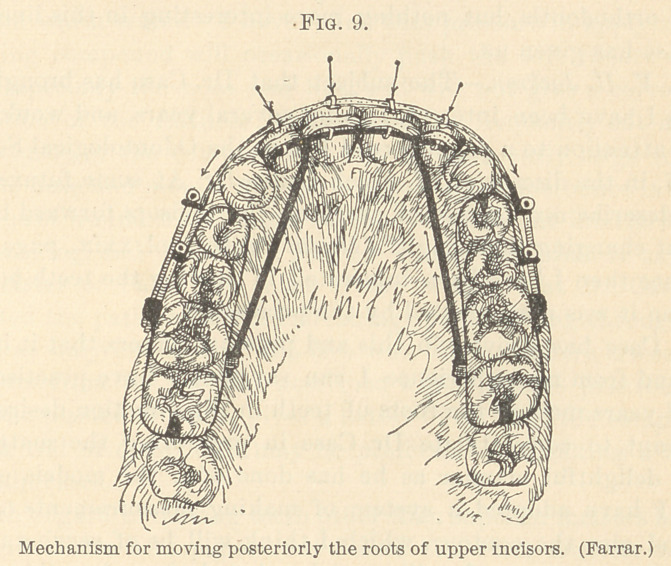


**Fig. 10. f10:**